# Accuracy of Patient-Specific Osteosynthesis in Bimaxillary Surgery: Comparative Feasibility Analysis of Four- and Two-Miniplate Fixation

**DOI:** 10.3390/jpm15050186

**Published:** 2025-05-04

**Authors:** Hylke van der Wel, Haye Glas, Johan Jansma, Rutger Schepers

**Affiliations:** 1 Clinic for Virtual Surgical Planning, 3D VSP B.V., Hoogwatum 2, 9906 TD Groningen, The Netherlands; 2 Department of Oral and Maxillofacial Surgery, Expertcenter for Orthofacial Surgery, Martini Hospital Groningen, Van Swietenplein 1, 9728 NT Groningen, The Netherlands

**Keywords:** bone plates, computer-aided design, orthognathic surgery, patient-specific computational modelling

## Abstract

**Background/Objectives**: Patient-specific osteosynthesis (PSO) plates, in combination with virtual surgical planning (VSP), have significantly improved the accuracy of orthognathic surgery. This study aimed to compare the surgical accuracy of two-plate versus four-plate fixation methods in Le Fort I osteotomies using PSO. **Methods**: A retrospective cohort study was conducted on 21 patients who underwent maxilla-first bimaxillary surgery at a single centre in 2024. Eight patients received two-plate fixation, while thirteen received four-plate fixation. All surgeries were planned using VSP. Postoperative cone beam computed tomography scans were used to assess the accuracy of maxillary positioning by comparing the planned versus achieved outcomes in terms of translation and rotation. **Results**: Both fixation methods yielded comparable results in maxillary positioning, with no significant differences observed between the two groups regarding translational or rotational deviations. The two-plate PSO approach demonstrated practical benefits, including reduced material usage and the potential for smaller surgical incisions, without compromising surgical accuracy. **Conclusions**: Two-plate PSO fixation is a viable alternative to the traditional four-plate method for Le Fort I osteotomies, offering similar accuracy with potential procedural advantages. While these findings support broader clinical adoption, further research is warranted to confirm the results in larger cohorts and to investigate biomechanical considerations.

## 1. Introduction

Since the introduction of rigid internal fixation, Le Fort I osteotomies have relied on rigid internal fixation using miniplates placed bilaterally at the nasomaxillary and zygomaticomaxillary buttresses [1]. This advancement replaced earlier techniques with wire fixation and intermaxillary fixation, offering greater stability. Traditionally, splint-based surgical techniques are used to guide maxillary positioning. This method has long been regarded as the gold standard for ensuring stability following maxillary repositioning. Over time, this approach has evolved with the adoption of patient-specific osteosynthesis (PSO), which facilitates the accurate transfer of surgical plans to the operating theatre, improving outcomes in terms of maxillary repositioning accuracy [2,3,4,5].

Subsequent to the transition from wire to rigid fixation and along with the introduction of patient-specific osteosynthesis, the concept of minimally invasive orthognathic surgery (MIOS) has emerged and gained prominence. A 2018 systematic review concluded that MIOS reduces postoperative morbidity and hospital stay compared with conventional approaches [6]. A subsequent 2024 narrative review confirmed these advantages, reporting lower intra-operative blood loss, shorter hospitalisation, and a faster return to function [7].

Although PSO is most commonly implemented with four customised fixation plates, the parallel rise in MIOS invites a re-evaluation of how much fixation plates are necessary. A number of studies have explored the use of two anteriorly placed fixation plates using traditional bendable osteosynthesis plates [8,9,10].

Mavili et al. demonstrated that fixation at the nasomaxillary buttress only provides sufficient stability for anteroposterior movements, challenging the necessity for additional fixation plates [10]. Susarla et al. supported this concept further, showing that two-plate fixation resulted in stable outcomes over a one-year period [8]. Similarly, Murray et al. reported no significant differences in postsurgical stability between two-plate and four-plate fixation methods [9]. These fundings suggest that, under certain conditions, two-point fixation may be adequate to achieve satisfactory skeletal stability, potentially simplifying surgical procedures and reducing tissue trauma. Despite these promising results, two-plate fixation remains relatively underexplored, particularly in combination with modern techniques like patient-specific osteosynthesis (PSO) and cutting guides.

Building upon this technological progress, three recent studies have combined PSO concepts with two-plate anterior fixation, proposing that it is a less invasive alternative to conventional four-point fixation [11,12]. Alfaro et al. introduced a novel plating system designed to enable a minimally invasive approach, highlighting potential benefits such as reduced operating time, lower surgical morbidity, and sufficient accuracy achieved through patient-specific implants [11]. Polido et al. presented their preliminary findings in an oral abstract, demonstrating that smaller patient-specific cutting guides and fixation plates can achieve accurate maxillary repositioning with smaller surgical incisions [12]. Amarista used a redesigned minimally invasive cutting-guide system to further demonstrate that careful guide anchorage preserves accuracy even through a limited canine-to-canine incision [13]. In addition to the clinical benefits, the use of two plates instead of four may lead to a reduction in material costs by reducing the used hardware to two plates [11].

The aim of the current study is to compare the accuracy outcomes of four-plate fixation and two-plate fixation methods for Le Fort I osteotomies performed using PSO. By analysing these two techniques, we aim to assess whether the two-plate anterior fixation translates into comparable accuracy We hypothesised that two-plate PSO fixation would result in comparable accuracy to four-plate fixation in Le Fort I osteotomies.

## 2. Materials and Methods

### 2.1. Study Design and Patient Selection

This study was designed as a retrospective cohort study. Patients operated on in the Martini Hospital Groningen in the year 2024 were identified by reviewing surgical records and virtual surgical planning (VSP) data from the specified time period. The 4-plate PSO system was applied before June 2024, whereafter a switch was made for the remainder of the year to the 2-plate PSO system. The inclusion and exclusion criteria are presented in Table 1. The study protocol was reviewed and approved by the Ethics Committee of the Martini Hospital Groningen (approval number 2025-006, dated 3 March 2025). Due to the retrospective nature of the study, the need for individual patient consent was waived, as confirmed by the committee.

### 2.2. Virtual Surgical Planning and PSO

All the operations were virtually planned by an experienced technical physician (HW or HG), using a pre-operatively made CBCT, Intra Oral dental scans. and patient photographs. VSP was performed with the help of the Materialise software (Materialise Enlight, version 5.0, Materialise NV, Leuven, Belgium). A consultation was held for every patient between the technical physician performing the planning and the surgeon performing the operation to refine the VSP. Subsequently, the surgeon reviewed the finalised operative plan with the patient to confirm agreement and obtain informed consent. Based on the agreed operative plan, patient-specific osteosynthesis was designed by the technical physician. During PSO design, screws were virtually positioned in regions of adequate bone thickness while avoiding dental roots and maintaining a safe distance between each screw hole and the planned osteotomy site.

**Figure 1:**
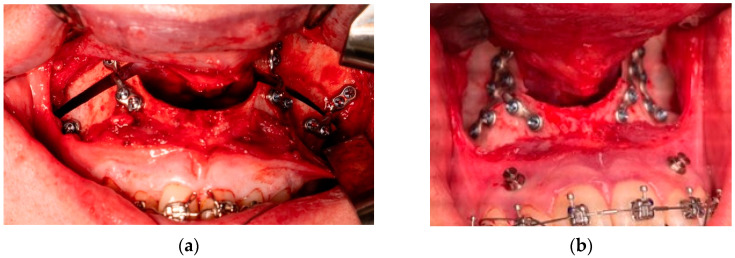
An overview of the two different PSO plating systems used: (**a**) 4-plate and (**b**) 2-plate PSO systems.

**Figure 2:**
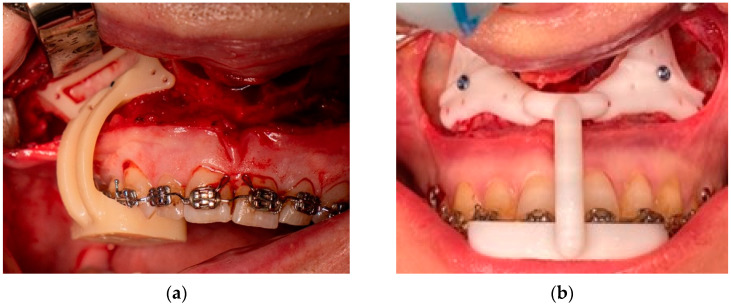
Comparison of the guide designs used for the (**a**) 4-plate and (**b**) 2-plate PSOs. Both guides are made using computer-assisted design and 3D printing, and both are tooth and bone borne. Both guides must be fixated using a fixation screw before using them to drill the screw holes.

Based on the design, the 4-plate PSO was manufactured by Createch Medical (Createch Medical SL, Mendaro, Spain), and the 2-plate PSO was produced by 3D VSP (3D VSP B.V., Bierum, The Netherlands) (see Figure 1). The guides for both PSO methods were designed using a combination of tooth-borne and bone-borne supports (see Figure 2). The guiding principle for the guides used for both PSO methods was the same, with the difference being that for the 2-plate PSO system, the guide could be placed through a smaller, more anterior incision. For the 4-plate system, two guides were used to drill the screw holes for both the left and right side. The final screw positions, guide designs, and plate designs were approved by the operating surgeon before production. Our design philosophy for the two-plate system incorporated a dorsal extension to facilitate additional screw fixation and a broader distribution of forces, without the need for additional dorsal plates. An intermediate splint was available for each patient as a backup in case a switch from PSO to splint-based surgery was necessary. A CAD/CAM splint was used for the final occlusion.

### 2.3. Surgery

Both the 2-plate and 4-plate PSO surgical fixation of the maxilla was performed following the maxilla-first protocol. Regarding the 2-plate PSO patients, the upper vestibular incision was extended from canine to canine, whereas the 4-plate fixation incision extended further towards the zygomaticomaxillary buttress. The plates were fixated with KLS Martin Maxdrive 1.5 mm screws (KLS Martin, Tuttlingen, Germany). Each patient’s mandibular translation and fixation were guided by the final dental splint. All surgeries were performed by one of two experienced oral and maxillofacial surgeons (JJ or RS).

### 2.4. Post-Operative Evaluation

Post-operative cone beam computed tomography (CBCT) images were acquired typically 7 to 10 days after the surgery, as part of the routine care protocol. The accuracy of the maxillary placement, relative to the pre-operative plan, was evaluated with the systematic image registration approach in the Materialise Mimics software (Materialise enlight v 5.0, Materialise, Leuven, Belgium). First, voxel-based registration was used to align the skull of the post-operative CBCT scan with the skull of the pre-operative CBCT scan. This alignment ensures that the skull is consistently positioned across the pre-operative, planning, and post-operative datasets. Once the skull was aligned, a second voxel-based registration was performed to align the maxilla to its post-operative position. This step isolated the movement and placement of the maxilla independent of the skull alignment. To quantify positional accuracy, cephalometric landmarks, identical to the landmarks used in the VSP, were used. The differences (Δ-values) between the planned and post-operative maxillary positions in 3 directions determined the accuracy. The delta values were calculated in the natural head position, which was pre-operatively determined, providing an accurate representation of the post-operative cephalometric deviations relative to the surgical plan.

### 2.5. Statistical Evaluation

Statistical analyses were conducted using IBM SPSS Statistics version 30 (IBM Corp., Armonk, NY, USA). The normality of the data was assessed using the Shapiro–Wilk test. As the data were not normally distributed, the Mann–Whitney U test was employed to assess the significant differences between the two groups. A *p*-value of less than 0.05 (*p* < 0.05) was considered to be indicative of statistical significance.

## 3. Results

### 3.1. Study Data

In total, 23 patients operated on in 2024 met the inclusion criteria. However, two patients were excluded from analyses because they did not undergo PSO. Among the included patients, eight received a two-plate PSO and thirteen a four-plate PSO. The demographics of the patients included for analyses are shown in Table 2. A Mann–Whitney U test was performed to compare ages between groups, showing a trend toward a difference that did not reach statistical significance (*p* = 0.058).

Table 3 presents the planned maxillary translations and rotations at the level of the upper incisor for both groups. The median values along with their interquartile ranges (IQR) are provided for each movement direction. There were no significant differences between the groups for any of the planned movements. 

### 3.2. Accuracy Results

As summarised in Table 4, no statistically significant differences were observed between the two-plate and four-plate PSO groups in terms of postoperative maxillary positioning accuracy. This was consistent across all three translational dimensions (anteroposterior, vertical, and transverse) as well as all rotational axes (pitch, roll, and yaw). While the data showed slightly higher median deviations in the two-plate group for certain parameters, these differences did not reach statistical significance.

## 4. Discussion

This study was enacted to compare the accuracy of two-plate and four-plate fixation methods for Le Fort I osteotomies performed with patient-specific osteosynthesis (PSO) in bimaxillary orthognathic surgery. While PSO has been widely recognised for its precision and ability to improve surgical outcomes, its application in two-plate anterior fixation has remained underexplored.

Our findings indicate that the accuracy of both two-plate and four-plate fixation were similar in terms of planned versus post-operative maxillary positioning. These findings are consistent with the Mavili et al. [10] and Susarla et al. [8] observations, using bendable plates, demonstrating that two-plate fixation provides adequate stability, particularly for anteroposterior displacements. While their focus was on stability rather than on immediate post-operative accuracy, the outcomes underscore the biomechanical sufficiency of anterior-only fixation in maintaining maxillary position. Additionally, Alfaro et al. [11] recently reported the feasibility of performing minimally invasive Le Fort I osteotomies using two- instead of four-plate PSOs. Their study’s accuracy was excellent, with mean deviations of less than 1 mm across key anatomical landmarks [11]. This reinforces the precision of PSO-based procedures and highlights our study’s contribution by demonstrating that the accuracy of two-plate fixation is comparable to four-plate fixation, thereby offering an alternative for Le Fort I osteotomies. Based on these findings, we accept our initial hypothesis that two-plate fixation provides comparable accuracy to four-plate PSO in the context of Le Fort I osteotomies.

Although our study primarily assesses the immediate postoperative accuracy of maxillary positioning, this metric alone does not fully reflect the overall clinical performance of the fixation methods. Notably, long-term stability, bone healing, and complication rates (e.g., hardware failure or relapse) were not evaluated in this analysis. While long-term follow-up results for the four-plate PSO system have been previously reported [14], future research should incorporate extended follow-up data for the two-plate method.

The reduction in the number of plates offers several practical advantages. First, it may reduce the material costs associated with PSOs, as fewer plates and possibly fewer screws are required [11]. Second, the two-plate approach makes the surgical procedure less invasive by requiring smaller incisions compared to the four-plate method. However, the biomechanical stability of two-plate fixation warrants further consideration. Essen et al. [15] demonstrated experimentally that two-plate fixation may not provide sufficient stability when the maxillary advancements exceed 10 mm. In contrast, Susarla et al. [8] reported that two-plate anterior fixation alone achieved stable outcomes with minimal relapses one year postoperatively. Yet, a finite element analysis (FEA) study comparing two-plate and four-plate fixation demonstrated that four-plate fixation reduces both the size and magnitude of the stress fields on the maxillary bone [16]. However, these studies employed conventional (pre-bent) plates with different plate designs. The Essen and Erkmen experimental studies utilised conventional L-plates with two screws per osteotomy side at the nasomaxillary buttress, whereas Susarla employed pre-bent plates with at least three screws per side, sourced from different manufacturers. Alfaro et al. [11], on the other hand, used specifically designed plates, with greater thickness and more screws, to optimise stability. In our study, the two-plate fixation method employed extended plates instead of conventional L-shaped fixation plates, which may have contributed to increased stability. Furthermore, our study focused exclusively on bimaxillary surgeries, where the mandible was also operated on. This combined surgical intervention possibly reduces the bite forces exerted on the maxilla during the early healing phase, potentially mitigating the risk of instability associated with two-plate fixation. Nonetheless, larger advancements may still necessitate additional support. As this study was designed as a feasibility analysis, it was not capable of detecting small effect sizes. Rather, it aimed to provide initial clinical evidence on the accuracy of two-plate PSO fixation. These findings will inform the design of future studies with larger cohorts and formal power calculations. Future studies should delineate the biomechanical limits of two-plate fixation, particularly under varying surgical conditions and for significant maxillary advancements or segmental Le Fort osteotomies. Although not statistically significant, the near-threshold *p*-value for planned roll (*p* = 0.060) suggests a possible trend that may reach significance in larger studies. This observation highlights the importance of further research with increased sample sizes to fully assess whether specific movement patterns, such as roll, are influenced by the number of fixation plates used.

This study has several limitations. First, the small sample size, particularly for the two-plate PSO group, limits the generalizability of our findings. The small sample size is due to the selected retrospective period, which was the year 2024. The reason for performing a retrospective analysis over a relatively short time period is that, in June 2024, our centre switched from four-plate PSOs to two-plate PSOs, allowing for a direct comparison of outcomes between the two methods within the same surgical environment. A second limitation is that the age distribution between the groups is notably different; however, there is currently no evidence to suggest that age influences the accuracy outcome. Thirdly, different manufacturers for the two- and four-plate systems may lead to variations in design and material properties, potentially affecting mechanical performance independently of the plate number. Consequently, these manufacturer-related differences may confound our comparisons and should be further investigated in more controlled studies. Finally, in the current analyses, segmental osteotomies were not included. As PSO is routinely used for segmental cases, future studies should explore the outcomes of two-plate fixation in this subgroup to provide a more comprehensive assessment. Finally, patient reported outcome measures were not collected in this study, although subjective parameters such as patient complaints could add valuable information and should be studied in future research.

## 5. Conclusions

The findings demonstrate that the accuracy of two-plate anterior fixation using PSO is comparable to the four-plate PSO method for Le Fort I osteotomies. The less invasive surgical approach, with possible reduced operating times and reduced materials, makes two-plate fixation a promising alternative. Further studies with larger sample sizes, long-term follow-up, and additional outcome measures, such as patient satisfaction and complication rate, are needed to fully validate the clinical utility of this technique, particularly in more complex cases such as segmental osteotomies.

## Figures and Tables

**Table 1:** Inclusion and exclusion criteria.

Inclusion Criteria	Exclusion Criteria
Patients underwent a bimaxillary osteotomy	Surgery not performed using PSO
Maxilla-first sequence used	
A one-piece Le Fort I was performed	

**Table 2:** Demographics of the patients included for analyses.

	Two-Plate PSO	Four-Plate PSO
Nr. of patients	8	13
Mean age (years)	25.4 ± 11	36.8 ± 12

**Table 3:** Planned maxilla translations and rotations in the two study groups—movement at the upper incisor; median values with interquartile range.

Direction	Two-Plate PSO	Four-Plate PSO	*p*-Value
	Median [IQR]	Median [IQR]	
Ant/post (mm)	6.8 (Ant) [4.4 (Ant)–7.9 (Ant)]	7.13 (Ant) [3.7 (Ant)–8.6 (Ant)]	0.913
Up/down (mm)	0.0 [1.5 (Down)–0.0 (Up)]	1.5 (Down) [2.1 (Down)–0.0 (Up)]	0.365
Left/right (mm)	0.0 [0.8 (R)–1.5 (L)]	0.0 [0.5 (R)–0.6 (L)]	0.766
Roll (°)	2.2 CCW [2.6 (CCW)–1.8 (CW)]	0.1 CCW [0.5 (CCW)–0.3 (CW)]	0.060
Pitch (°)	2.7 (CCW) [4.0 (CCW)–0.4 (CW)]	2.5 (CCW) [6.1 (CCW)–3.0 (CW)]	0.856
Yaw (°)	0.0 [0.9 (CCW)–1.8 (CW)]	0.1 CW [1.5 (CCW)–2.9 (CW)]	0.828

Abbreviations: Ant, anterior; CCW, counterclockwise; CW, clockwise; IQR, interquartile range; L, left; PSO, patient-specific osteosynthesis; R, right.

**Table 4:** Absolute deviations from the planned position of the maxilla in the two study groups—deviation at the upper incisor; median values with interquartile range.

	Two-Plate PSO	Four-Plate PSO	*p*-Value
	Median [IQR]	Median [IQR]	
Anterior/posterior (mm)	1.4 [0.7–2.2]	1.3 [0.4–2.8]	0.885
Up/down (mm)	1.6 [0.6–2.1]	0.9 [0.5–1.6]	0.277
Left/right (mm)	0.9 [0.2–1.6]	0.2 [0.2–0.6]	0.059
Roll (°)	1.0 [0.4–1.2]	0.4 [0.3–0.9]	0.119
Pitch (°)	2.4 [0.4–3.0]	1.5 [0.7–4.9]	0.515
Yaw (°)	1.1 [0.5–1.5]	0.6 [0.5–0.8]	0.096

IQR, interquartile range; PSO, patient-specific osteosynthesis.

## Data Availability

Data are available on request due to privacy/ethical restrictions.
